# Supplementary material for: Multiscale characterization reveals oligomerization dependent phase separation of primer-independent RNA polymerase nsp8 from SARS-CoV-2
Source: Commun Biol. 2022 Sep 7;5:925. doi: 10.1038/s42003-022-03892-x (PMC9451113; doi:10.1038/s42003-022-03892-x)
Supplement: Supplementary file 2 — Supplementary Material [file 42003_2022_3892_MOESM2_ESM.pdf]

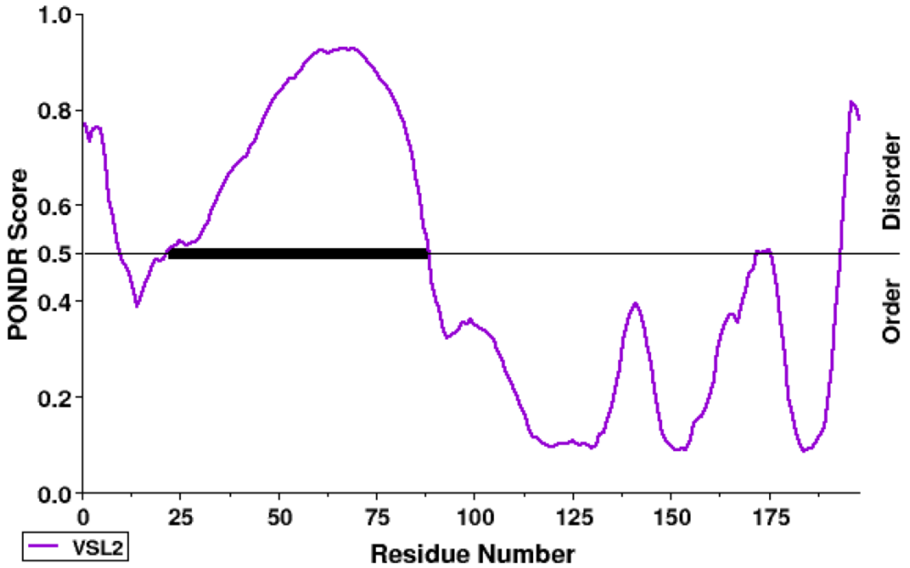

Supplementary Figure 1 Intrinsic disorder region (IDR) analysis of SARS-CoV2 nsp8 with PONDR software (http://www.pondr.com/). Black bar indicates potential IDR.

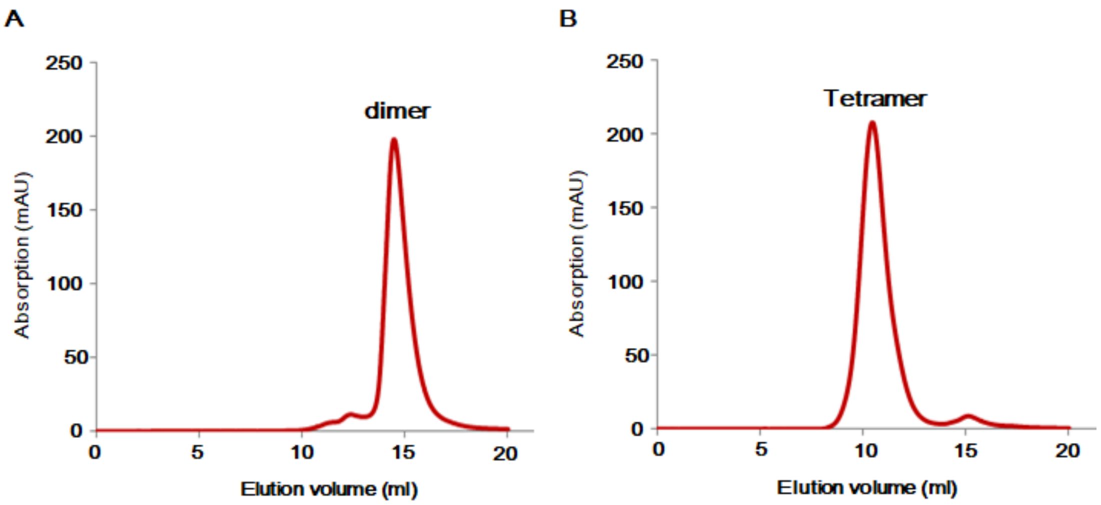

Supplementary Figure 2 Dimer (A) and tetramer (B) form nsp8 were analyzed with superdex increase 200, respectively, again standing for 10 days. Before loading sample, superdex increase 200 was equilibrated with buffer containing 20 mM HEPES pH 7.4, 0.5 M NaCl, 1mM DTT.

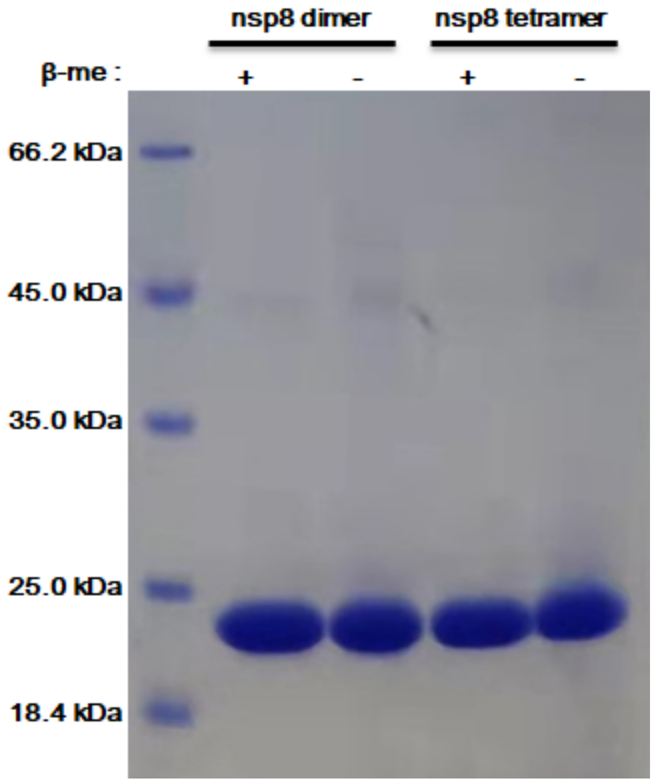

Supplementary Figure 3 nsp8 dimer or tetramer at concentration of 0.5 mg/ml dissolved in buffer 20 mM HEPES pH 7.4, 0.5 M NaCl, was mixed with loading buffer with or without 0.7 M β-mercaptoethanol (β-me) and analyzed by SDS-PAGE. The uncropped gel is available in Supplementary Data 2.

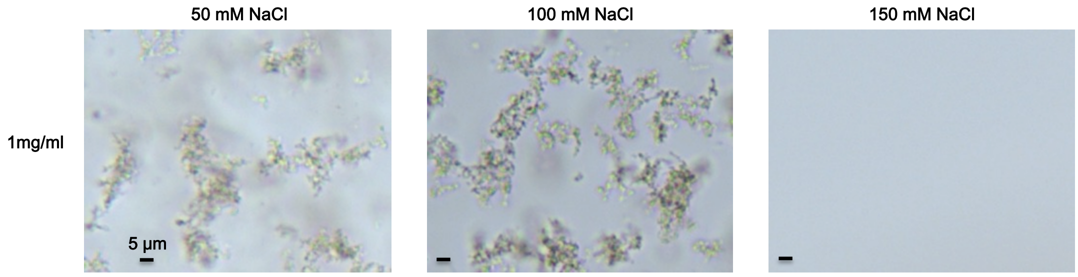

Supplementary Figure 4 nsp8 tetramer forms solid-like structure at low NaCl concentration (100 mM or lower).

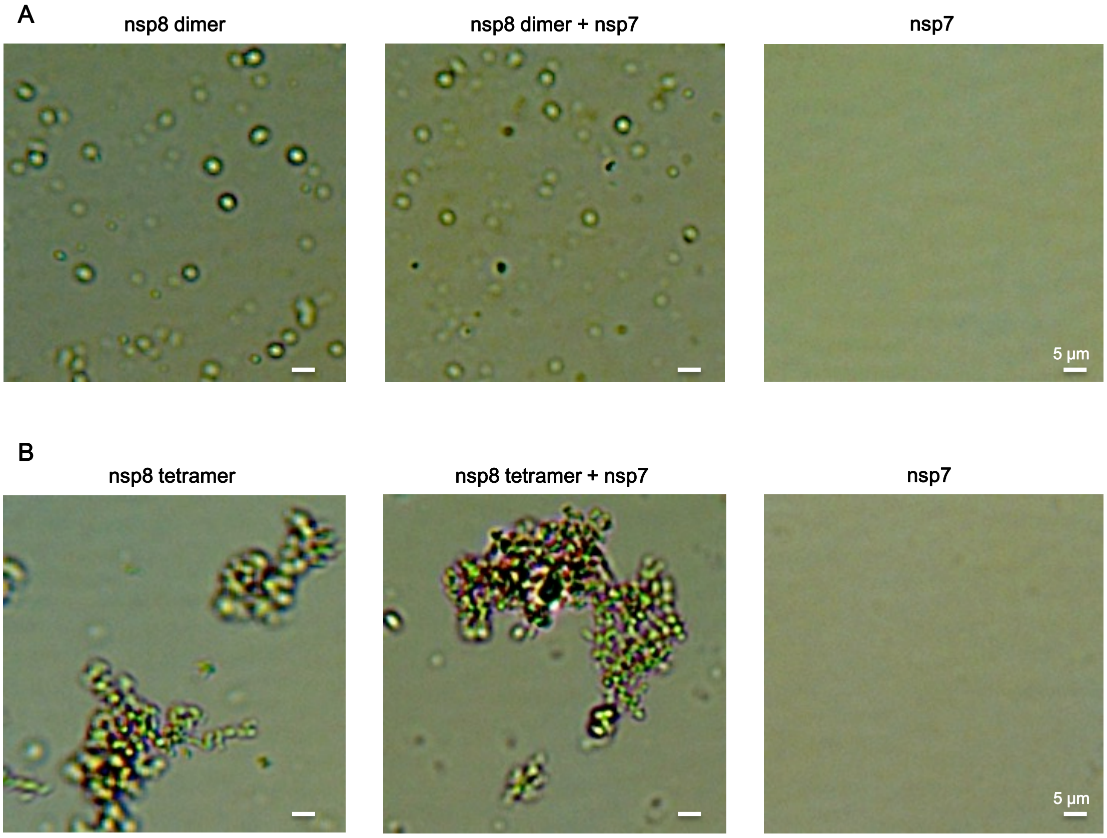

Supplementary Figure 5 Phase transformation behavior of nsp8 can't be affected by mixing with nsp7. (A) Differential interference contrast (DIC) imaging of nsp8 dimer (2 mg/ml), mixture of nsp8 dimer (2 mg/ml) and nsp7 (1 mg/ml), and nsp7 (1 mg/ml), dissolved in buffer 20 mM HEPES pH 7.4, and 100 mM NaCl. (B) Differential interference contrast (DIC) imaging of nsp8 tetramer (1 mg/ml), mixture of nsp8 tetramer (1 mg/ml) and nsp7 (0.5 mg/ml), and nsp7 (0.5 mg/ml), dissolved in buffer 20 mM HEPES pH 7.4, and 100 mM NaCl.

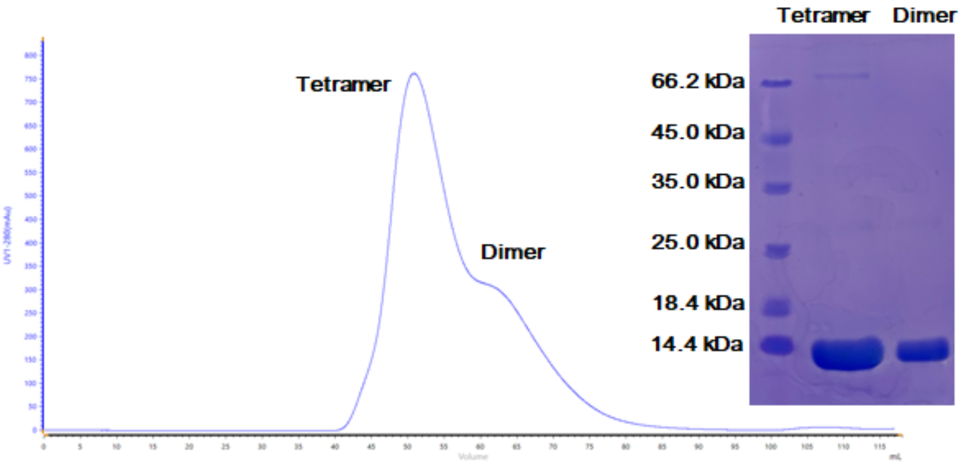

Supplementary Figure 6 Purification of Nsp8ΔN76 with gel filtration. The uncropped gel is available in Supplementary Data 3.
