# Supplementary material for: Multiscale characterization reveals oligomerization dependent phase separation of primer-independent RNA polymerase nsp8 from SARS-CoV-2
Source: Commun Biol. 2022 Sep 7;5:925. doi: 10.1038/s42003-022-03892-x (PMC9451113; doi:10.1038/s42003-022-03892-x)
Supplement: Supplementary file 3 — Description of all Supplementary Data [file 42003_2022_3892_MOESM3_ESM.pdf]

# Description of Additional Supplementary Files

**File name:** Supplementary Data 1
**Description:** The source data for the graphs presented in the figures of this paper.

**File name:** Supplementary Data 2
**Description:** Uncropped SDS-PAGE gel for Supplementary Figure 3.

**File name:** Supplementary Data 3
**Description:** Uncropped SDS-PAGE gel for Supplementary Figure 6.
