# Supplementary figures and images for: Multiscale characterization reveals oligomerization dependent phase separation of primer-independent RNA polymerase nsp8 from SARS-CoV-2
Source: Commun Biol. 2022 Sep 7;5:925. doi: 10.1038/s42003-022-03892-x (PMC9451113; doi:10.1038/s42003-022-03892-x)

The uncropped SDS-PAGE gel of Supplementary Figure 3.


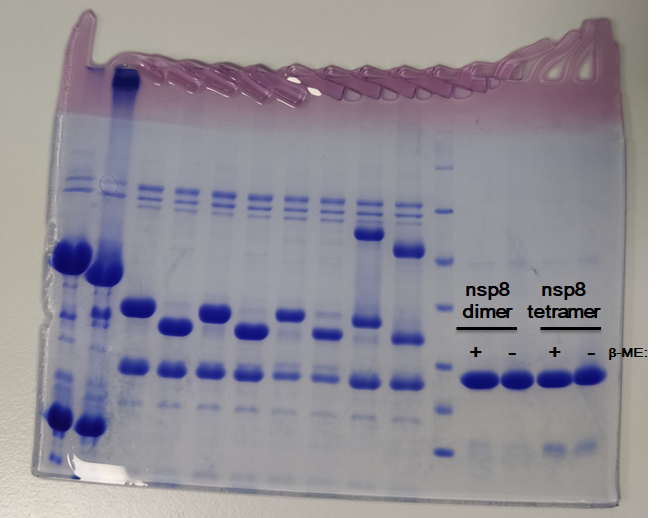

Supplement: Supplementary file 5 — Supplementary Data 2 [file 42003_2022_3892_MOESM5_ESM.docx]

The uncropped SDS-PAGE gel of Supplementary Figure 6.


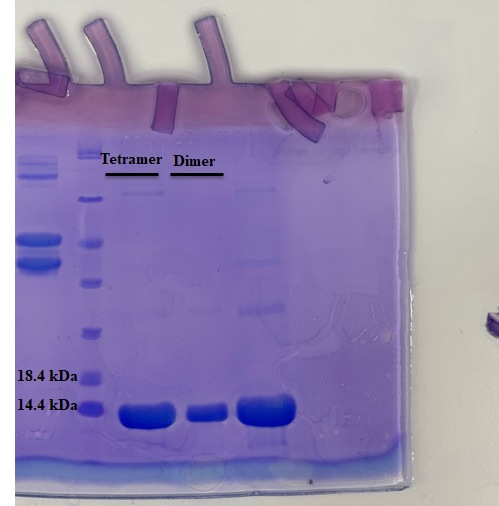

Supplement: Supplementary file 6 — Supplementary Data 3 [file 42003_2022_3892_MOESM6_ESM.docx]
